# Psychotherapeutic Intervention in the Demobilization Process: Addressing Combat‐related Mental Injuries with Narrative Exposure in a First and Second Dissemination Stage

**DOI:** 10.1002/cpp.1986

**Published:** 2015-12-16

**Authors:** Anke Köbach, Susanne Schaal, Tobias Hecker, Thomas Elbert

**Affiliations:** ^1^ Department of Psychology University of Konstanz Konstanz Germany; ^2^ vivo international Konstanz Germany; ^3^ Department of Psychology University of Ulm Ulm Germany; ^4^ Department of Psychology University of Zürich Zürich Switzerland

**Keywords:** PTSD, aggression, demobilization, treatment, dissemination, ex‐combatants

## Abstract

**Background:**

Depending on the exposure to traumatic stressors and combat, 20% to 50% of ex‐combatants present with trauma‐related disorders, and more than half of the members of armed groups have a proclivity to violence. Therefore, psychotherapeutic assistance should address both, trauma‐related suffering and the lowered threshold for aggressive behaviour.

**Objective:**

Supporting the demobilization process of ex‐combatants in the eastern DR‐Congo, we implemented a version of Narrative Exposure Therapy adapted for Forensic Offender Rehabilitation (FORNET).

**Method:**

In two successive dissemination stages (DS), local counsellors conducted FORNET. In DS1, they were trained by clinical experts, and in DS2, the by then experienced counsellors trained and supervised a second group of local counsellors (DS2). The training consisted of a 3‐week workshop covering theoretical concepts and practical therapeutic skills. In DS1 and DS2, a total of 98 demobilizing combatants received an intervention; treatment‐as‐usual served as the control condition. Posttraumatic stress disorder, appetitive aggression, depression severity and drug dependence were assessed prior to the intervention and 6 and 12 months later; additionally, we assessed reintegration success.

**Results:**

Six months post‐intervention, FORNET significantly reduced Posttraumatic stress disorder symptoms but had less effect on the trait of appetitive aggression; moreover, beneficial effects were found for depression severity and drug dependence as well as for reintegration indices. Treatment gains were retained at 12 months.

**Conclusions:**

Individuals without previous training in psychotherapy can learn to effectively apply the brief intervention FORNET and support the demobilization process in ongoing conflicts. The study suggests that it is possible to pass down psychotherapeutic techniques over generations of counsellors. © 2015 The Authors. *Clinical Psychology & Psychotherapy* published by John Wiley & Sons Ltd.

**Key Practitioner Message:**

- Posttraumatic stress symptoms, depression and clinically relevant levels of drug dependence can effectively be reduced with a version of Narrative Exposure Therapy (NET) adapted for Forensic Offender Rehabilitation (FORNET).
- The intervention is effective in the context of ongoing conflict.
- Individuals without previous training in psychotherapy can learn to effectively apply the brief intervention FORNET.
- It is possible to pass down psychotherapeutic techniques like FORNET over generations of counsellors.
- Psychotherapeutic interventions like FORNET may facilitate the transition to peace in war‐torn regions.

About 20% to 50% of ex‐combatants in post‐conflict regions suffer from mental health complications. Posttraumatic stress disorder (PTSD), depression, suicidal ideation and/or substance use disorders are highly prevalent (Betancourt *et al*., 2013; Heltemes, Clouser, MacGregor, Norman, & Galarneau, 2014; Johnson *et al*., 2008, 2010; Nock *et al*., 2013; Odenwald *et al*., 2007; Walker, 2010). In addition, ex‐combatants present with tendencies towards aggressive behaviour. These enhanced violent reactions are particularly associated with the hypervigilance symptom cluster of (combat‐related) PTSD (Jones, 2012; Klostermann, Mignone, Kelley, Musson, & Bohall, 2012; MacManus, Dean, & Jones, 2013; Morland, Love, Mackintosh, Greene, & Rosen, 2012). Subsequent to the experience of combat high (Grossman, 1995, p. 243; Köbach, Schaal, & Elbert, 2015; Köbach *et al*., in press), ex‐combatants did however also reported a shift towards perceiving self‐exerted violence as appealing (appetitive aggression; e.g., Elbert, Weierstall, & Schauer, 2010; Weierstall, Schaal, Schalinski, Dusingizemungu, & Elbert, 2011; Hecker, Hermenau, Maedl, Elbert, & Schauer, 2012; Weierstall *et al*., 2013; Crombach & Elbert, 2014a). Current research in neuroscience indicates that appetitive aggression follows distinct neural pathways in comparison with the aforementioned PTSD‐related or reactive aggressiveness (Moran, Weierstall, & Elbert, 2014).

The implementation of psychotherapeutic interventions into demobilization programmes faces two critical challenges. First of all, there is an enormous lack of adequately trained personnel in low‐ and middle‐income countries (LMIC), where current demobilization programmes are situated. Therefore, shifting tasks from clinical experts to trained counsellors has emerged as a key strategy to encounter the problem (Jacob, Neuner, Maedl, Schaal, & Elbert, 2014; Saxena, Thornicroft, Knapp, & Whiteford, 2007). However, clinical trials were rather heterogeneous yet (Tol *et al*., 2013). Considering a general population, the evidence of passing down psychotherapeutic techniques to non‐mental health professionals in LMIC is analysed in Van Ginneken *et al*. (2013). They conclude that the overall evidence of psychotherapeutic interventions addressing PTSD and related disorders is still ‘low’ (Van Ginneken *et al*., 2013). Promising effects were mainly found for cognitive therapy approaches (e.g., Prolonged Exposure (PE), Cognitive Processing Therapy (CPT), or Narrative Exposure Therapy (NET); Foa *et al*., 2005; Bass *et al*., 2013; Jacob *et al*., 2014; Neuner *et al*., 2008). In addition to the lack of potential personnel, it seems that psychotherapeutic interventions are not yet perfectly tailored for the specific group of (ex‐)combatants, or military personnel. Most importantly, the effect sizes of front‐line trauma interventions are lower than that could be expected from results reported in other samples. In a recent review, Steenkamp and Litz (2013) have reported that treatment effects for the reduction of Posttraumatic stress in veterans and active military personnel were only moderate. For NET, Stenmark, Guzey, Elbert, and Holen (2014) demonstrated that effects were smaller in ex‐combatants than in those who have never held a combatant status. Potential reasons for the reduced effect may include appetitive perceptions of violence (Elbert *et al*., 2010; Köbach *et al*., 2015), anger (Morland *et al*., 2012; Forbes *et al*., 2008; Forbes, Creamer, Hawthorne, Allen, & McHugh, 2003) and/or feelings of guilt (Kubany, 1994).

These war‐related changes are not ‘only’ restricted to the subjects' clinical suffering. They are associated with various forms of psychological malfunctioning that affect the individuals' minds, bodies, families and communities (Schauer & Schauer‐Kaiser, 2010). In consequence, the combatants' history of war and violence appears to interfere with reintegration and peacekeeping efforts (for review, see Maedl, Schauer, Odenwald, & Elbert, 2010; Walker, 2010), and thus, ex‐combatants bear the risk of becoming a major source of destabilization (Banholzer, 2014). What had been figured in the outline of historical incidents of (groups of) ex‐combatants who resorted to violence in the past, is mirrored in the psychological field research. In Northern Uganda, Vinck, Pham, Stover, and Weinstein (2007) found that attitudes towards violent conflict resolution were associated with stronger symptoms of PTSD and depression. Particularly ex‐combatants present with an enhanced ability of perceiving aggression as intrinsically rewarding (Elbert *et al*., 2010; Köbach *et al*., 2015). Such a positive perception of self‐committed violence has retrospectively been demonstrated to predict repeated re‐enlistment after demobilization (Hermenau, Hecker, Mädl, Schauer, & Elbert, 2013; Elbert *et al*., 2013). Furthermore, Ertl, Pfeiffer, Schauer‐Kaiser, Elbert, and Neuner (2014) demonstrated a positive association between the exposure to war, mental illness and maladjustment in a sample of Ugandan child soldiers. Savoca and Rosenheck (2000) and Gear (2002) found elevated unemployment rates in US veterans and in former combatants in South Africa, respectively. A number of researchers have demonstrated high stability of the aforementioned psychological complications in similar populations (Milliken, Auchterlonie, & Hoge, 2007; Pigeon, Campbell, Possemato, & Ouimette, 2013; Thomas *et al*., 2010).

In the last decade, some trials have investigated the effectiveness of psychotherapeutic assistance in post‐conflict regions (e.g., Ertl, Pfeiffer, Schauer, Elbert, & Neuner, 2011; McMullen, O'Callaghan, Shannon, Black, & Eakin, 2013; Bass *et al*., 2013). One of the best established approaches in these demanding settings is NET (Schauer, Neuner, & Elbert, 2005, 2011). Schauer *et al*. (2011) have developed NET as a short‐term intervention aimed at reducing PTSD symptoms resulting from exposure to multiple traumatic events. The intervention comprises 8–12 individual sessions of approximately 90 min, is manualised, brief, disseminable and culturally sensitive. NET takes into account the whole range of lifetime exposure to traumatic events, each within the context of the client's biography (Schauer *et al*., 2011). Evidence for the effectiveness is summarized in Schauer *et al*. (2011) and includes randomized controlled trials with between‐group designs, which demonstrated superiority in comparison with active (e.g., Bichescu, Neuner, Schauer, & Elbert, 2007; Catani *et al*., 2009; Hensel‐Dittmann *et al*., 2011; Ertl *et al*., 2011; Neuner, Schauer, Klaschik, Karunakara, & Elbert, 2004) and inactive (e.g., Crombach & Elbert, 2014b; Hermenau, Hecker, Schaal, Mädl, & Elbert, 2013; Adenauer *et al*., 2011; Ruf *et al*., 2010) control conditions with regard to the reduction of PTSD severity/diagnosis and related disorders. Cohen's *d* effect sizes are large. Adenauer *et al*. (2011) showed that NET induces changes in functional brain organization, and Morath, Gola, Sommershof *et al*. (2014) and Morath, Moreno‐Villanueva, Hamuni *et al*. (2014) demonstrated beneficial outcome with molecular biological measures (T‐cell distribution and DNA strand break accumulation). Clinical trials were conducted in refugee settings in Germany, Norway and Uganda and in community samples in China, DR Congo, Germany, Japan, Romania, Rwanda, Saudi Arabia and Sri Lanka. Moreover, dissemination trials with lay counsellors providing NET are implemented effectively since 2008 (Neuner *et al*., 2008; Jacob *et al*., 2014). For reviews and meta‐analysis, see inter alia Robjant and Fazel (2010), Crumlish and O'Rourke (2010) and Gwozdziewycz and Mehl‐Madrona (2013). In this article, we provide a brief practical guideline for NET in the Method/Interventions Section; the main manual published in English (and translated also to Korean, Dutch, French, Italian, Japanese and Slovak) describes the procedure in more detail (Schauer *et al*., 2011).

As other current front‐line trauma interventions (e.g., PE, CPT and other trauma‐focused therapies; Difede, Olden, & Cukor, 2014), NET is theoretically based on the maladaptive formation of memory components in the aftermath of the traumatic event, resulting in a highly cohesive ‘trauma network’ (Brewin, Gregory, Lipton, & Burgess, 2010; Schauer *et al*., 2011). However, having been a warrior does also implicate the experience of combat high (Grossman, 1995, p. 243; Köbach *et al*., 2015). The associative memory thereof may include similar sensory cues as for purely traumatic experiences, but this time connected to cognitions and emotions with a positive valence. Combatants thus develop what has been termed ‘hunting network’ (Elbert *et al*., 2010), an associative network of memory representations related to combat events, that connects sensory and perceptual information (e.g., the sight of blood, the sound of cries and victory and the smell of fire) with the memories for cognitive (e.g., the thought ‘I will be the hero’), affective (e.g., feelings of anger, joy and excitement) and physiological responses (e.g., increase in heart rate, increased muscular strength and reduced pain sensitivity) that were elicited during the events. When this propositional network is recalled, mind and body become aroused and braced for actions such as fighting or killing (Elbert *et al*., 2010). In other words, resorting to often extreme forms of violence may be a strategy of (ex‐)combatants to associate cues like blood with the hunting network rather than with traumatic memories in order to cope with fear and trauma. It is noteworthy, that with its rewarding properties, violence has the potential for a process addiction, as sex, food, stimulants or gambling does (Schneider, Irons, & Physicians, 2001; APA, 2013).

Conceptualized as coping, trait (Weierstall & Elbert, 2011), or addiction, appetitive forms of aggression need to be addressed in course of the demobilization process.

In response, Hecker, Hermenau, Crombach, and Elbert (2015) adapted NET for patients with a history of perpetrated violence, thus for Forensic Offender Rehabilitation (FORNET). FORNET aims at re‐organizing pathological memory formations and targets memories of traumatic stress and combat high in addition. Furthermore, FORNET focuses on the role change from a combatant to a civilian; interpersonal therapy in a group setting had been shown to effectively improve functioning in Uganda (Bolton *et al*., 2007) and Rwanda (Schaal, Elbert, & Neuner, 2009). In a first randomized controlled trial, Hermenau *et al*. (2013) demonstrated that FORNET successfully reduced PTSD symptoms and facilitated reintegration by decreasing the connection to (para)military life in former child soldiers from the DRC. Moreover, Crombach and Elbert (2014b) showed that FORNET reduced the number of criminal acts performed by former street children in Burundi.

The goal of the present study was to investigate the effectiveness of FORNET delivered by local counsellors trained in a first and second dissemination stage. We expect that ex‐combatants who receive FORNET, compared with treatment‐as‐usual (TAU), will show a greater reduction in Posttraumatic stress and appetitive aggression (primary outcome) as well as lower depression and drug dependence (secondary outcome). In addition, we aimed at examining the course or reintegration (economic reintegration and connection with (para)military life; secondary outcome). Moreover, we hypothesize that FORNET conducted by local non‐health professionals that were trained by experts will be effective (dissemination stage 1), even when conducted by local non‐health professionals who have been trained by experienced peer‐counsellors (dissemination stage 2). Finally, we aim to examine the course of the treatment outcome in a subsample. We predict that treatment gains will be long lasting and observable in 1‐year follow‐ups.

## Methods

### Trial Design

The trial had two successive dissemination stages (DS): in the first stage, local individuals without previous experience in psychotherapy were trained by clinical experts (DS1). In the second stage, the by then experienced counsellors from the first stage trained a second group of local individuals (DS2). DS1 and DS2 incorporated three phases of treatment delivery; each DS featured a balanced parallel group, semi‐random design with a TAU control condition.

### Participants

**Figure 1:**
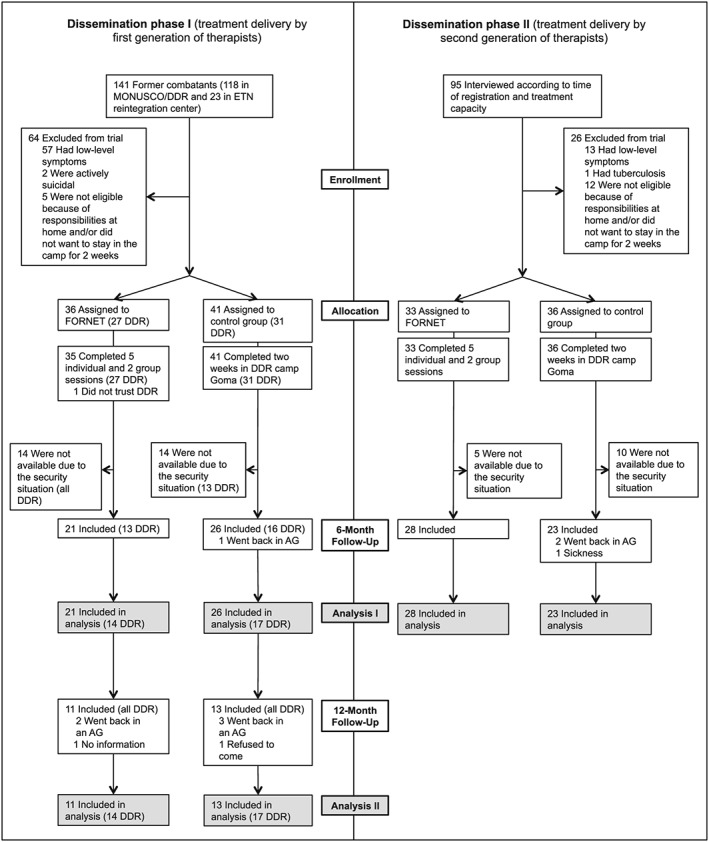
Flow of participants. DDR = disarmament, demobilization and reintegration programme (MONUSCO)

We included only adult male combatants from the eastern Democratic Republic of Congo (DRC) suffering from trauma symptoms and heightened levels of aggression (≥7 symptoms of PTSD and ≥7 items affirmed in the aggression questionnaire). Exclusion criteria were serious physical injury or sickness and acute suicidal ideation or psychosis. The final sample contained a total of 98 male former combatants with a M = 23.48 (SD = 5.81). Table 1 describes the sample characteristics at baseline per treatment condition; DS1 and DS2 did not differ regarding these variables. The flow of participants is described in Figure 1.

**Table 1:** Sample characteristic at trial baseline divided by treatment condition and dissemination stage

		FORNET		TAU	
	DS	M ± SD	[CI]	M ± SD	[CI]
Age in years	1	21.90 ± 4.74	[19.75, 24.06]	23.31 ± 5.35	[21.15, 25.47]
	2	23.14 ± 5.52	[21.00, 25.28]	25.52 ± 7.18	[22.42, 28.63]
Years of education	1	5.43 ± 3.56	[3.81, 7.04]	6.77 ± 3.62	[5.31, 8.23]
	2	5.57 ± 3.94	[4.04, 7.10]	4.35 ± 3.59	[2.80, 5.90]
Age in years of entry in first AG	1	16.05 ± 6.47†	[13.02, 19.08]†	16.46 ± 5.09	[14.04, 18.52]
	2	17.79 ± 5.65	[15.59, 17.98]	18.96 ± 6.15	[16.30, 21.62]
Month spent in the AG	1	44.97 ± 33.95†	[29.09, 60.86]†	46.62 ± 52.81	[25.29, 67.95]
	2	50.11 ± 45.62	[32.42, 67.80]	57.80 ± 52.45	[35.12, 80.48]
Lifetime traum. events	1	14.19 ± 2.66	[12.98, 15.40]	12.88 ± 2.98	[11.67, 14.09]
	2	14.04 ± 2.63	[13.02, 15.06]	13.57 ± 3.13	[12.21, 14.92]
Lifetime perp. violence	1	5.43 ± 2.42	[4.33, 6.53]	3.96 ± 2.07‡	[3.11, 4.81]‡
	2	5.32 ± 1.93	[4.15, 5.33]	4.74 ± 1.36	[4.57, 6.07]

*Note:*

* *p* ≤ 0.05,

** *p* ≤ 0.01,

*** *p* ≤ 0.001. DS1: *n*_FORNET_ = 21, *n*_TAU_ = 26; DS2: *n*_FORNET_ = 23, *n*_TAU_ = 28;

† *n* = 20;

‡ *n* = 25;

DS = dissemination stage. AG = armed group. FORNET = Narrative Exposure Therapy for Forensic Offender Rehabilitation. TAU = treatment‐as‐usual. SD = standard deviation. CI = confidence interval.

### Setting

The trial took place at the MONUSCO (Mission de l'Organisation des Nations Unies pour la Stabilisation en République Démocratique du Congo) demobilization camp and in a reintegration centre run by a local, non‐governmental, non‐profit organization, both settled in Goma, the capital of the North Kivu province in the eastern DRC.

#### Democratic Republic of Congo

After a history of war and corruption, the DRC is currently struggling to recover from one of the most brutal wars of the recent decades (e.g., British Broadcasting Corporation (BBC) country profile). A century ago, the vast country with its immense economic resources was subject to one of the most brutal colonial occupations on historical record by the Belgian King Leopold II (1877–1908) and the Belgian parliament (1908–1960). Following this, Colonel Joseph Desire Mobutu (later Mobutu Sese Seko) established a three‐decade‐long authoritarian regime (1965–1997; Zaire since 1971). The eastern parts of the DRC in particular were subject to intertribal conflicts throughout history (Richard, 2013). In the 1990s, the conflict escalated with the Rwandan genocide. A huge wave of refugees, including Hutu genocidaires, migrated into the Kivu regions. In reaction, Rwandan and Ugandan armies (with support from Angola, Burundi and Tanzania) entered the eastern DRC launching the First Congo War (1996–1998), immediately followed by the Second Congo War, also known as Africa's Great War (1998–2002). These wars caused one of the most severe human disasters of this century (Stearns, 2011). Although fighting continues today (Elbert *et al*., 2013), the signing of the Lusaka Peace Accord in 1999 brought the conflict formally to an end. The same year, a United Nations (UN) mission (MONUC, since 2010 MONUSCO) was deployed to the DRC. With the raise the insurgency Mouvement Mars 23 (M23) in April 2012, the eastern DRC encountered another period of high conflict intensity that peaked when the rebels took over the regions' capital Goma. After a conjoint counter strike of the national army and the therefore implemented forced intervention brigade of the MONUSCO, the rebels withdrew from their occupied territories in November 2013.

#### MONUSCO/Disarmament, Demobilization, Reintegration

Disarmament, demobilization, reintegration (DDR) of Congolese armed groups had been implemented as a core component of the peace mission in 2001 (UNDPKO, 2010). Today, DDR in the eastern DRC has grown into the most expensive and long‐lasting mission of the UN (Allen, 2011; Richard, 2013).

Presently, combatants who wish to officially demobilize join the DDR facilities closest to their base where they hand over their arms. Afterwards, they are brought to one of the larger, centralized DDR camps, either in Goma (North Kivu) or Bukavu (South Kivu), where they exchange their uniforms for civilian clothing and receive official demobilization documents. In this period, ex‐combatants have access to a medical doctor and counsel from DDR personnel. For Congolese ex‐combatants, there were no further services in terms of vocational training and etc. at the time of the data collection (July 2012–October 2013).

In 2013, DDR processed 1595 adult combatants and 464 child soldiers. In total, 1050 weapons and 42 373 rounds of ammunitions were recovered and destroyed. The majority of ex‐combatants demobilized from Forces Démocratiques de Libération du Rwanda, M23 and various Mai‐Mai groups, which are a sort of local paramilitary (pseudo‐)defence networks (MONUSCO, 2014).

All participants of the present study spent approximately 2–3 weeks on site to receive the intervention.

#### Reintegration Centre

In addition to the UN‐led DDR programme, there are local and international non‐governmental organizations that provide assistance to former (child) soldiers. One of these organizations is the reintegration centre where we conducted a part of the study. Here, ex‐combatants receive a 1‐year vocational training; the setting is comparable with that of boarding schools where the learners live apart from their homes, partly in foster families, and acquire the chosen profession (carpenter, mechanic and tailor). Ex‐combatants in this reintegration centre are also provided with a social worker for any kind of personal problems.

### Procedure

Soldiers willing to demobilize attended the camp daily. They usually arrived in groups and were registered by the screening unit in an apparently random order. At each stage of dissemination, we randomized the first half of eligible participants to FORNET and TAU on the basis of their registration sequence (restricted randomization with 1:1 allocation ratio; uneven numbers FORNET, even numbers TAU). The second half of participants was then matched to the randomized participants according to their PTSD symptom severity and level of aggression. The matched clients received the opposite treatment of their counterpart. In the reintegration centre, we proceeded likewise. In the process of data analysis, participants without a follow‐up assessment, mostly due to security risks (see Figure 1 Flow of participants), were excluded. This number was unexpectedly high, and therefore, we dropped the matching dyads. In the final sample, approximately half of participants (53.1%) were randomized. The matched participants were allocated in order to balance symptom severity in the two groups and to facilitate the flow of treatments. This occurred without knowing any personal characteristics that could possibly influence the outcome. While all authors had worked in Goma, the first author was on site during the entire training and assessment period. The first author also implemented the random allocation and constantly monitored the trial (including data assessment and treatment phases). None of the screening unit, the counsellors or the interviewers was able to observe, to foresee or to influence the allocation process.

In the first days after their arrival, we interviewed all adult male ex‐combatants who arrived at the DDR camp between 23 until 29 July, 7 until 17 August and 30 August until 15 September 2012 (DS1), or 2 until 11 February, 27 February until 13 March and 26 March until 5 April 2013 (DS2). The assessments were stopped once each counsellor had 1–2 eligible clients. Ex‐combatants in the reintegration centre were selected by the director and interviewed at the centre. Follow‐up assessments were conducted in February, March and April 2013 (DS1: 6‐month follow‐up) and for both phases in August and September 2013 (DS1: 12‐month follow‐up; DS2: 6‐month follow‐up). The interviews were conducted individually in a private setting and lasted between 1.5 and 2.5 h. None of the subjects rejected participation in the primary diagnostic interview.

Diagnostic interviews (including inter‐rater, *n*_interviewer_ = 58) were carried out by a group of six mother‐tongue Kiswahili‐speaking research assistants (aged 21–31 years; 5 men, 1 woman) recruited from non‐MONUSCO/DDR settings (one psychologist, four psychology students and one translator) to ensure absolute blindness to the treatment conditions in the follow‐up assessments. During an intensive 10‐day workshop, these interviewers were trained in psychological concepts and in sensitive and empathetic interviewing techniques. They received follow‐up training sessions in October 2012 (3 days) and in February 2013 (7 days). During the entire phase of data collection, the interviewers received extensive feedback and were closely supervised by clinical experts. Three of the interviewers (the psychologist and two of the students) had other occupational duties when we started the DS2.

Prior to the start of the trial, independent interpreters translated all instruments in written form from English to Kiswahili, the lingua franca of the Kivu regions. A blind back‐translation in English ensured valid translation. Inconsistencies were discussed with both translators and a clinical expert.

After the interview, eligible participants were briefed on the general experimental set‐up: voluntarily participation, possibility for withdrawal at any time without negative consequences and compensation with a basic needs kit (including a mattress, a plastic sheet and a blanket). To keep the outcome expectations constant, the treatment was communicated as a talking programme and the control condition as a leisure programme for rest after an exhausting time in the military.

The Ethical Review Board of the University of Konstanz, the board of the NGO vivo international, the authorities of MONUSCO and the local reintegration centre approved the present study.

### Local Team of Counsellors and Dissemination Strategy

The majority of counsellors (17 in total) had worked in the DDR unit of MONUSCO and were selected to participate in the training by their superiors. We also included three staff members of the local reintegration centre (DS1) and two medical doctors (DS2) who worked for associated institutions. In two FORNET trainings (7 h/5 days/3 weeks), the theoretical concepts of PTSD, aggression and other trauma‐related disorders, as well as the treatment approach were taught theoretically and practically; particular focus was given to practical therapeutic skills in treating trauma and aggression using exposure in sensu. In addition, the trainees learned basic counselling skills. In the first and the second training courses, identical didactic tools were applied: interactive lecturing for theoretical content and a variety of practical exercises including role‐playing in front of the group by trainers and trainees, as well as in small groups (2–3 trainees) with and without supervision.

#### Dissemination Stage 1

To disseminate FORNET to the first generation of counsellors, two post‐doctoral clinical psychologists from the University of Konstanz conducted the initial 3‐week FORNET training. A clinical psychologist closely monitored and supervised (individually and in the group) the succeeding treatments. In the final part of the DS1 treatment phase, four counsellors were chosen to participate in the following train‐the‐trainer training as aspirant FORNET trainers.

#### Dissemination Stage 2

To disseminate FORNET to the second generation of counsellors, the four FORNET trainers conducted the second 3‐week FORNET training. First, they prepared their own training manual and scheduled the course of the training. Next, they carried out the training with their peers and received extended feedback from two clinical psychologists supervising the training. Finally, they provided regular peer supervision with trainees who conducted FORNET. The peer supervision was in turn supervised individually and in groups by the clinical psychologist on site.

Fidelity first to the treatment and second to the peer‐supervision protocol was ensured by a close supervision of the counsellors (at least twice a week plus one organizational meeting) and the peer ‐supervisors (at least once a week plus occasional sit‐ins). Both expert‐supervision (DS1) and peer‐supervision (DS2) followed the same protocol of case‐specific and model‐specific guidance of the counsellors.

### Interventions

#### Narrative Exposure Therapy

Over the last decade, Schauer *et al*. (2011) have developed NET as an evidence‐based short intervention for victims of multiple traumatization. Throughout the NET sessions, the client constructs a detailed chronological narration of his or her own biography. The first session comprises two parts: psychoeducation and the lifeline exercise. The latter aims at gaining an overview of the central events of the client's life story. In the exercise, the client situates stones and flowers along a rope beginning at birth and ending at the present day with an outlook to the future; stones represent negative/traumatic events (e.g., accident, natural disaster and abuse) and flowers major positive events (e.g., wedding and graduation). In the subsequent exposure sessions, the most traumatic experiences are re‐lived (emotional, sensory, cognitive and interoceptive) in order to achieve habituation of the fear reactions as well as a transformation of the generally fragmented report of traumatic events into a coherent narrative. To this end, the therapist records the event, and at the beginning of the next session, the narration is read to the patient and corrected for potential discrepancies. In the last session, the participant receives a written report of their biography that is read to them before both the therapist and the client sign the testimony.

In several trials, NET has effectively reduced trauma symptoms in four to six therapeutic sessions only (Neuner *et al*., 2008, 2004; Schaal *et al*., 2009).

#### Narrative Exposure Therapy for Forensic Offender Rehabilitation

In response to the shortcomings outlined in the introduction, Elbert, Hermenau, Hecker, Weierstall, and Schauer (2012) upgraded NET to address the pathological sequelae of combat high in addition to traumatic stress by means of narrative exposure, and furthermore, encountered the often difficult transition from combatant to civilian using interpersonal therapy in a group setting.

The intervention consisted of five individual and two group sessions. As in NET, the first session begins with the lifeline. In addition to the stones and flowers, the individual has also the opportunity to place sticks along the rope to symbolize active involvement in violent acts (e.g., combat, rape and massacre). This is performed in an attempt to avoid emotional valence or moral judgment being associated with such events. However, the client can combine a stick with a flower or a stone as a way to designate a positive or negative perception of the violent experience. In the exposure sessions, both the most traumatic experiences and the violent acts that involved the strongest emotions are re‐experienced in sensu. Here, the therapist focuses on the first perpetrated killing/severe injury and/or the first rape. During exposure sessions, the therapist encourages the client to re‐live the event and continually asks for current and past emotional, physiological/interoceptive, cognitive and sensory reactions, as in NET. However, after a narration of the first killing/injury and/or first rape, particular focus is given to the threshold the client overcame to harm the person/s and the diminution of this threshold in subsequent acts. Concluding the event, the client is encouraged to articulate current thoughts and feelings about the incident. Following the individual sessions, the two group sessions (1.5–2.5 h) consist of two therapists and 4–5 clients. Focusing on the role change from combatant to civilian, the clients are encouraged to discuss positive and negative aspects of the two roles and finally to frame their future hopes and wishes.

The adaptations of NET for the present study can be summed as follows: (1) the number of exposure sessions: 5 instead of 8–12 sessions; (2) an additional symbol for the lifeline: a stick representing active involvement in violent acts (e.g., combat, rape and massacre) in an attempt to avoid emotional valence or moral judgment being associated with such events; (3) a mandatory exposure in sensu to the first killing/injury, first rape/sexual assault and other major perpetrated act(s) (if present in the lifeline) with particular focus to the inhibitory threshold the client overcame to harm the person(s) and the diminution of this threshold in subsequent acts and current thoughts and feelings about the event; to this end, the counsellors were instructed to re‐tell a short summary of the exposed perpetrated act emphasizing the threshold the client overcame to harm the victim. Afterwards, the client was asked what she or he thinks about the act now (‘What do you think about it now?’). In case of an act of sexual violence, the client is additionally encouraged to consider consensual intercourse for the future (‘Why would people want to have consensual sexual intercourse? Why would you?’); (4) no written narration; and (5) the two final group sessions (1.5–2.5 h) that consist of two therapists and 4–5 clients.

Up until now, there have been two randomized controlled trials of FORNET: Hermenau *et al*. (2013) found FORNET to reduce PTSD symptoms and closeness to military life in a sample with former Congolese child soldiers of a reintegration centre in Goma, North Kivu, eastern DRC; Crombach and Elbert (2014b) demonstrated that former street kids in Burundi who received FORNET committed fewer violent acts compared with those in a waiting list control condition. Importantly, no evidence of harm was found, and no adverse effects were reported.

#### Treatment‐as‐usual

Participants assigned to TAU remained in the demobilization camp for 2–3 weeks or completed the usual programme in the local reintegration centre (1‐year training in manual trades), respectively: in the demobilization camp, they received medical care and had access to psycho‐social support (usually case‐specific advise to the best of the advisor's knowledge). In the reintegration centre, also medical care was offered, and social workers provided assistance in the form of counselling session(s); here, the problems of the client are heard and discussed in several meetings. The total amount of time the patients spent with the counsellor ranged from 2 to 15 h and more very few cases.

### Outcome Measures

The battery was administered as a semi‐structured interview in Kiswahili except for a few cases that called for Kinyarwanda. At the beginning of the interview, sociodemographic information was obtained from each participant and included age, ethnicity, educational background and details regarding the participant's military career.

#### Exposure to Violence

A 31‐item event‐checklist adapted from previous studies in similar populations (e.g., Hecker *et al*., 2012, 2013) was administered to assess lifetime exposure to different types of potentially traumatic events (experienced and witnessed) and perpetrated violent acts. A sum score was calculated for the number of types of witnessed and experienced traumatic events (possible range: 0–22) as well as for the number of types of perpetrated violent acts (possible range: 0–9). Reliability measures for the applied event‐checklist revealed high inter‐rater reliability (Cohen's *κ* = 0.98). For further details, see Köbach *et al*. (2015).

#### Primary Outcome

Participants' diagnostic status and PTSD symptom severity were evaluated using the PTSD Symptom Scale‐Interview (PSS‐I; Foa & Tolin, 2000). The PSS‐I assesses the 17 Diagnostic and Statistical Manual of Mental Disorders, 4th Edition DSM‐IV APA, 2000 symptom criteria for PTSD. Each item is rated on a four‐point scale ranging from 0 (*not at all*/*only once*) to 3 (*five or more times per week*/*almost always*). The diagnostic status was determined by counting the number of symptoms endorsed (≥1) per each symptom cluster (one for re‐experience, three for avoidance and two for arousal) as well as significant distress. We measured the symptom occurrence during the previous month (instead of the last 2 weeks) to address the given duration in the DSM (criterion F: the symptoms were encountered during the last 4 weeks). PTSD severity was calculated by summing all of the symptom scores (possible range 0–51). Internal consistency and inter‐rater reliability revealed excellent values (Cronbach's *α* = 0.91; intraclass correlation coefficient, ICC = 0.89).

Appetitive aggression was assessed using the Appetitive and Facilitative Aggression Scale (AFAS; DS1) and the Appetitive Aggression Scale (AAS, Weierstall & Elbert, 2011; DS2 and follow‐ups). The AAS has been shown to have excellent psychometric properties but does not assess reactive aggression. Therefore, we initially applied the AFAS, which revealed low variance for our African sample for DS1. The original version of the AFAS contains 30 items that assess current appetitive (e.g., ‘Was it so fascinating to beat someone up, that you could not stop fighting?’) and reactive (‘facilitative’, e.g., ‘Were you so irritated, that you took it out on other people?’) aggression, and the AAS consists of 15 items assessing appetitive aggression only (e.g., ‘Once fighting has started, do you get carried away by the violence?’). Several items asked by the AAS and the AFAS to assess the level of appetitive aggression yield to evaluate the same sub‐concepts (e.g., not caring for one's integrity during a fight: ‘When you fight, do you stop caring about whether you could be killed?’; the according AFAS item: ‘Was fighting so much fun, that you did not care if you were hurt or not?’). Both instruments rate the statements on a five‐point scale; notably, the AFAS is on a frequency scale from *never* (0) to *more than two times a week* (4), whereas the AAS is on a consent scale from *I disagree* with the given question (0) to *I totally agree* (4). The appetitive aggression score we used in the final analysis consisted of seven dichotomous items covering the facets that were assessed in both questionnaires: (1) testing strength against others is fun; (2) seeing the victim suffering or (3) bleeding as well as (4) harming others is exciting; (5) rush of fighting; (6) habituating to cruelty; and (7) not caring for one's integrity during a fight. The statements were considered true if the according item of the AFAS or AAS, respectively, was approved. Psychometric properties were excellent (ICC = 0.93).

#### Secondary Outcome

Diagnostic status and depression symptom severity were determined using the Patient Health Questionnaire‐9 (Kroenke & Spitzer, 2002). The nine items correspond to the DSM‐IV symptom criteria for Major Depression and assess the participants' feelings 2 weeks prior to testing. Each item is rated on a four‐point scale ranging from 0 (*not at all*) to 3 (*nearly every day*). The diagnostic status was determined if at least five symptoms were endorsed, including one of the cardinal symptoms (depressive mood, loss of interest). In this sample, the Cronbach's *α* coefficient was 0.86 and indicated excellent inter‐rater reliability (ICC = 0.96).

Drug Dependence was diagnosed according to DSM‐IV symptom criteria using the Texas Christian University Drug Screen II (Knight, Simpson, Janis, & Morey, 2002), a standardized 9‐item tool that assesses (yes/no) each criterion according to the participant's behaviour in the past 12 months. For the 6‐month follow‐up assessment, we asked for the last 6 months. The severity of drug dependence was calculated by summing up all of the items (possible range: 0–9). The diagnostic status was determined if at least three symptoms were endorsed. In evaluations, the instrument has demonstrated stability across racial and ethnic subgroups (Simpson, Joe, Knight, Rowan‐Szal, & Grey, 2012). Its psychometric properties are highly satisfactory (Cohen's *κ* = 0.94).

To evaluate the clients' reintegration success, we assessed economic status and the participants' connection with (para)military life. The outstanding importance of sustaining military networks in the aftermath of war with regard to the risk of re‐engagement in organized violence is analysed in Nilsson (2008). Economic reintegration was assessed by a selection of five questions; each answer was converted into a point system from which the total score was computed (Table 2). The index ranges from 0 (failed economic reintegration) to 30 (highly successful economic reintegration). The inter‐rater reliability was excellent (Cohen's *κ* = 0.93). Furthermore, we utilized 12 items to assess the connection with (para)military life. We asked questions about the frequency of actual contact with armed forces and about the subject's affiliation with the specific lifestyle and attitudes of (para)military groups. Each item was rated on a scale from 0 (*not at all*) to 4 (*more than two times a week*/*very much*) and added up to a final score ranging from 0 to 60. Table 2 shows the list of items and their descriptive statistics. The internal consistency was good for the 6‐month follow‐up assessment (Cronbach's *α* = 0.81) but poor for the 12‐month follow‐up (Cronbach's *α* = 0.20). The inter‐rater reliability was excellent (ICC < 0.99) for the instrument at both time points.

**Table 2:** Descriptive information on economic reintegration and connection with (para)military life 6 months after demobilization

	DS1	DS2	
Economic reintegration			Points
Do you have a job currently? Yes (n, %)	40 (85.1)	40 (78.4)	0/5
How much is your regular income ($)? (M, ±SD)	3.21 (3.74)	4.66 (3.99)	0–10
Do you have your own house? Yes (n, %)	11 (23.4)	18 (35.3)	0/5
Do you have your own mobile phone? Yes (n, %)	17 (36.2)	17 (33.3)	0/5
How often do you eat meat or fish per week? (M, ±SD)	2.25 (1.63)	1.78 (1.57)	0–5
Connection with (para)military life (yes/≤1)	n (%)	n (%)	Scale
How often where you in contact with your former unit/commander, any other armed group?	21 (45.7)	9 (17.6)	0‐5
How often did your former unit/try to convince you to go back to an armed group?	27 (58.7)	7 (13.7)	0‐5
How often did any other armed group try to convince you to go back to an armed group?	18 (39.1)	12 (23.5)	0‐5
How often were you in contact with former combatants who engaged in criminal activities?	4 (8.7)	1 (2.0)	0‐5
How often did you engage in criminal activities?	4 (8.7)	0 (0)	0‐5
How often did you get problems with the police?	6 (13.0)	11 (21.6)	0‐5
How often did you get arrested?	7 (15.2)	7 (13.7)	0‐5
How often did you think about going back to an armed group?	11 (15.2)	7 (13.7)	0‐5
How often did you make specific plans to join an armed group again?	6 (13.0)	3 (5.9)	0‐5
How much did you consider yourself as a soldier?	15 (33.3)	12 (23.5)	0‐5
How much did you enjoy to talk about your life as soldier?	25 (54.3)	25 (49.0)	0‐5
How much did you enjoy to think back about your life as soldier?	26 (56.5)	24 (48.0)	0‐5

*Note:* DS = dissemination stage.

### Analysis

Group differences were calculated using the *Chi*‐square test for categorical and the *t*‐test (independent and paired; one‐tailed for directional hypotheses) for continuous variables. Additionally, we report 95% confidence intervals (CI). Bonferroni adjustment of 95% significance levels specifies the *p*‐value at *p* ≤ 0.025 for primary and *p* ≤ 0.0125 for secondary outcomes. Cohen's *d* effect sizes between 0.20 and 0.49 indicate a small effect, 0.50 and 0.79 a medium effect and ≥0.80 a large effect. To test the efficacy of FORNET, we conducted 2 (treatment condition) × 2 (DS) × 2 (time: baseline, 6‐month follow‐up) mixed‐model analyses of covariance (ANCOVA) for PTSD and appetitive aggression (primary outcome) and for trauma‐related syndromes (depression and drug dependence); baseline symptom severity and setting (DDR reintegration centre) were entered as covariates (Analysis I; Figure 1). Likewise, 2 (group) × 3 (time: baseline, 6‐month follow‐up, 12‐month follow‐up) repeated measures ANCOVAs (baseline symptom severity as covariates) were conducted to confirm the maintenance of treatment gains (Analysis II; Figure 1). Deviations from normal distribution and heteroscedasticity were not found to influence the result's robustness. As the sub‐samples had equal sizes, the ANCOVAs could be regarded as robust against violations of homogeneity of variances.

Eta squared effect sizes—partial ( $\varepsilon_{p}^{2}$) and generalized ( $\varepsilon_{G}^{2}$)—were computed; generalized ( $\varepsilon_{G}^{2}$) allows comparisons across different (repeated measure/mixed model) AN(C)OVA designs (Bakeman, 2005; Lakens, 2013; Olejnik & Algina, 2003). Our metric for a small effect was *ε* ^2^ ≥ 0.0099, for a medium effect *ε* ^2^ ≥ 0.0588 and for large effect *ε* ^2^ ≥ 0.1379 (Cohen, 1988). To estimate possible differences in efficacy for DS1 and DS2, we calculated the confidence intervals for Cohen's *d* for both stages. We considered Cohen's *d* effect sizes within the FORNET condition to be equivalent when there was a substantial match (>80%) between DS1 and DS2. Furthermore, we checked the three‐way interaction term (group × time × dissemination stage) for insignificance. In the 6 months follow‐up test, we lost some of the matched participants (*n* = 42) due to the deterioration in the security situation. To maintain the sample size, we did not require exact matching dyads in the final analysis. Inter‐rater interviews (*N* = 77; *n*_expert_ = 19, 24.7%) were conducted throughout the whole assessment process. Following Hallgren (2012), we computed ICCs (two‐way, mixed; absolute agreement) for ordinal and Cohen's *κ* for nominal data. We used spss 21 and R (version 2.15.0) for the analysis.

## Results

**Figure 2:**
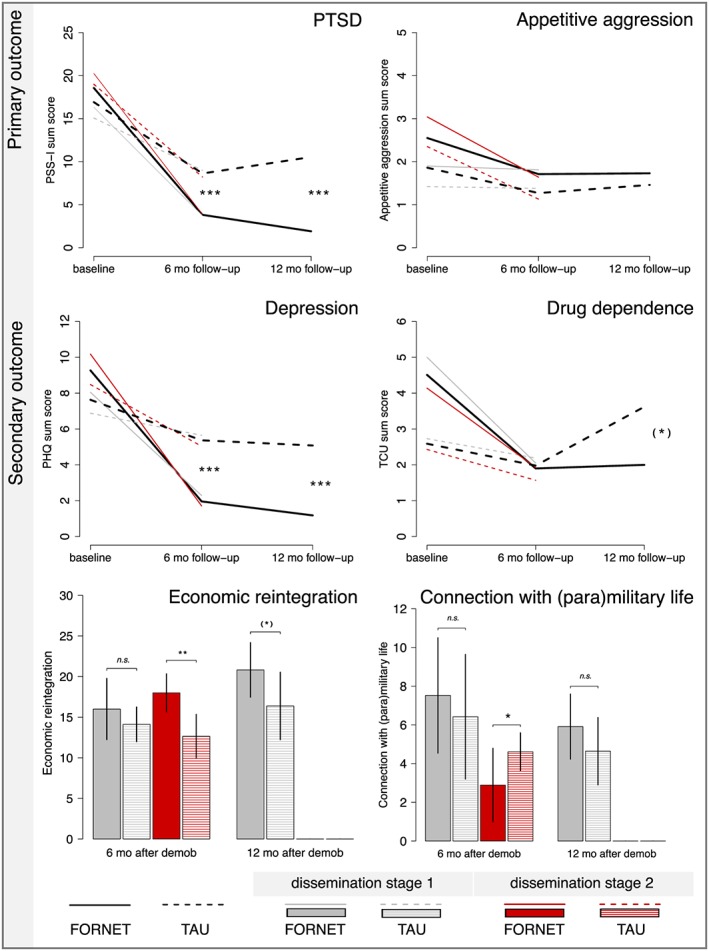
Treatment gains for primary (PTSD, appetitive aggression) and secondary (depression, drug dependence, economic reintegration, connection to (para)military life) outcome measures. Note: Interaction effects for 6‐month and 12‐month follow‐up assessments, including the total sample of Analysis I (Figure 1) at baseline and 6‐month follow‐up and, complementary, the sample of Analysis II at 12‐month follow‐up. ^(*)^ *p* ≤ 0.10, ^*^ *p* ≤ 0.05, ^**^ *p* ≤ 0.01, ^***^ *p* ≤ 0.001. FORNET = Narrative Exposure Therapy for Forensic Offenders Rehabilitation. TAU = treatment‐as‐usual. DS = dissemination stage. [Colour figure can be viewed at wileyonlinelibrary.com]

Baseline exposure to violence is summarized in Table 1. Descriptive statistics for primary and secondary outcome measures divided by treatment condition and DS are displayed in Table 3. An overview of the results is provided in Figure 2. Within‐group comparisons are reported in the results, subsection Dissemination. Baseline, 6‐month and 12‐month follow‐up for FORNET and TAU condition divided by DS1 and DS2.

**Table 3:** Baseline, 6‐month and 12‐month follow‐up scores for FORNET and TAU condition divided by DS1 and DS2

		FORNET		TAU			
	DS	M ± SD	[CI]	M ± SD	[CI]	Statistic	Cohen's *d* [CI]
Primary outcome measures							
PTSD severity							
Baseline	1	16.33 ± 8.59	[12.42, 20.24]	15.08 ± 6.91	[12.29, 17.87]	*t*(45) = −0.56	−0.16 [−0.76, 0.44]
	2	20.25 ± 9.76	[16.47, 24.03]	19.00 ± 8.24	[15.44, 22.56]	*t*(49) = −0.49	−0.14 [−0.71, 0.44]
6 mo after demob	1	3.71 3 ± 4.71	[1.54, 5.89]	9.00 ± 8.28	[5.66, 12.34]	*t*(45) = 2.74**	0.76 [0.13, 1.38]
	2	3.89 ± 6.34	[1.44, 6.35]	8.22 ± 6.95	[5.21, 11.22]	*t*(49) = 2.32**	0.65 [0.06, 1.25]
12 mo after demob	1†	1.19 ± 3.08	[−0.16, 3.98]	10.54 ± 10.31	[4.31, 16.77]	*t*(22) = 2.67**	1.09[0.14, 2.05]
Appetitive aggression							
Baseline	1	1.90 ± 2.19	[0.91, 2.90]	1.42 ± 1.70	[0.74, 2.11]	*t*(45) = −0.85	−0.25[−0.86, 0.36]
	2	3.04 ± 1.90	[2.30, 3.77]	2.35 ± 1.82	[1.56, 3.14]	*t*(49) = −1.31	−0.37[−0.95, 0.21]
6 mo after demob	1	1.81 ± 2.06	[0.87, 2.75]	1.38 ± 1.88	[0.63, 2.14]	*t*(45) = −0.74	−0.23[−0.82, 0.39]
	2	1.64 ± 1.89	[0.91, 2.38]	1.13 ± 1.06	[0.67, 1.59]	*t*(49) = −1.16	−0.33 [−0.91, 0.25]
12 mo after demob	1†	1.73 ± 1.27	[0.87, 2.58]	1.46 ± 1.71	[0.43, 2.50]	*t*(22) = −0.43	−0.17 [−1.06, 0.72]
Secondary outcome measures							
Depression severity							
Baseline	1	8.05 ± 4.58	[5.96, 10.13]	6.88 ± 5.82	[4.53, 9.24]	*t*(45) = −0.75	−0.21 [−0.83, 0.39]
	2	10.18 ± 4.91	[8.28, 12.08]	8.48 ± 5.07	[6.29, 10.67]	*t*(49) = −1.21	−0.34 [−0.92, 0.24]
6 mo after demob	1	2.29 ± 3.80	[0.56, 4.01]	5.65 ± 4.87	[3.69, 7.62]	*t*(45) = 2.60**	0.76 [0.14, 1.39]
	2	1.71 ± 3.35	[0.41, 3.01]	5.04 ± 5.64	[2.61, 7.48]	*t*(49) = 2.62**	0.74 [0.14, 1.33]
12 mo after demob	1†	1.18 ± 1.98	[−0.09, 2.45]	5.08 ± 4.87	[2.13, 8.02]	*t*(22) = 2.49**	1.02 [0.07, 1.97]
Drug dependence							
Baseline	1	5.00 ± 3.10	[3.59, 6.41]	2.73 ± 3.07	[1.49, 3.97]	*t*(45) = −2.51**	−0.74 [−1.36, 0.11]
	2	4.14 ± 3.35	[2.84, 5.44]	2.43 ± 3.01	[1.13, 3.74]	*t*(49) = −1.89*	−0.53 [−1.12, 0.05]
6 mo after demob	1	2.05 ± 2.58	[0.87, 3.22]	2.19 ± 2.48	[1.19, 3.19]	*t*(45) = 0.20	0.06 [−0.55, 0.66]
	2	1.93 ± 2.75	[0.86, 2.99]	1.57 ± 2.63	[0.43, 2.70]	*t*(49) = −0.48	−0.13 [−0.71, 0.44]
12 mo after demob	1†	2.00 ± 2.19	[0.53, 3.47]	3.62 ± 3.15	[1.71, 5.52]	*t*(22) = 1.42^(*)^	0.59 [−0.32, 1.49]
Reintegration: socioeconomic reintegration							
6 mo after demob	1	16.00 ± 8.63	[12.07, 19.93]	14.12 ± 5.41	[11.93, 16.30]	*t*(45) = −0.91	−0.27 [−0.87, 0.33]
	2	18.00 ± 6.18	[15.61, 20.39]	12.65 ± 6.46	[9.86, 15.45]	*t*(49) = −3.01**	−0.84 [−1.45, −0.25]
12 mo after demob	1†	20.82 ± 5.56	[17.08, 24.56]	16.38 ± 7.49	[11.85, 20.91]	*t*(22) = −1.62^(*)^	−0.66 [−1.58, 0.25]
Reintegration: connection with (para)military life							
6 mo after demob	1	7.52 ± 6.81	[4.42, 10.62]	6.42 ± 8.19	[3.11, 9.73]	*t*(45) = −0.49	−0.14 [−0.75, 0.46]
	2	2.89 ± 2.57	[1.90, 3.89]	4.61 ± 4.58	[2.63, 6.29]	*t*(49) = 1.69*	0.47 [−0. 11, 1.06]
12 mo after demob	1†	5.91 ± 2.88	[3.97, 7.84]	4.62 ± 3.01	[2.79, 6.44]	*t*(22) = −1.07	−0.21 [−1.10, 0.67]

*Note:*

* *p* ≤ 0.10,

* *p* ≤ 0.05,

** *p* ≤ 0.01,

*** *p* ≤ 0.001. AG = armed group. mo = month. demob = demobilization. DS = dissemination stage. FORNET = Narrative Exposure Therapy for Forensic Offender Rehabilitation. TAU = treatment‐as‐usual; DS1: *n*_FORNET_ = 21, *n*_TAU_ = 26; DS2: *n*_FORNET_ = 28, *n*_TAU_ = 23;

† DS1: *n*_FORNET_ = 11, *n*_TAU_ = 13.

### Treatment Outcome

#### Primary Outcome

Mixed‐model ANCOVA showed a significant interaction of time × treatment (*F*(1, 92) = 14.15, *p* ≤ 0.001, $\varepsilon_{p}^{2}$ = 0.133, $\varepsilon_{G}^{2}$ = 0.095), indicating greater PTSD symptom reduction in the FORNET group compared with TAU. The main effect of time did not reach significance. Between‐group comparisons 6‐month post‐treatment showed significantly fewer symptoms in the FORNET condition compared with TAU (*t*(96) = 3.55, *p* < 0.001, Cohen's *d* = 0.72). Between‐group effect sizes were moderate. The remission rate of ex‐combatants who received FORNET was substantially higher compared with TAU: at baseline, 29 ex‐combatants in the FORNET group fulfilled the diagnostic criteria for PTSD; 6 months later, two‐thirds (*n* = 19, 66%) were in remission. In TAU, a total of 23 participants suffered from PTSD at baseline, and at 6 months later, 10 (43%) were in remission. The number of former combatants suffering from the clinical symptoms of PTSD at the 6‐month follow‐up differed significantly among the two groups (*χ* ^2^(1) = 5.77, *p* = 0.015).

No significant effects were found with regard to the level of appetitive aggression (interaction effect of time × treatment: *F*(1, 92) = 1.13, *p* = 0.291, $\varepsilon_{p}^{2}$ = 0.012, $\varepsilon_{G}^{2}$ = 0.008, main effect of time: *F*(1, 92) = 0.04, *p* = 0.947, $\varepsilon_{p}^{2}$ < 0.001, $\varepsilon_{G}^{2}$ < 0.001). Accordingly, between‐group comparisons were also not significant (*t*(96) = −1.27, *p* = 0.104, Cohen's *d* = −0.26).

#### Secondary Outcome

A mixed‐model ANCOVA for the severity of depression showed a significant interaction of time × treatment (*F*(1, 92) = 14.87, *p* ≤ 0.001, $\varepsilon_{p}^{2}$ = 0.139, $\varepsilon_{G}^{2}$ = 0.097). The main effect of time did not reach the 95% significance level (*F*(1,92) = 3.33, *p* = 0.071, $\varepsilon_{p}^{2}$ = 0.035, $\varepsilon_{G}^{2}$ = 0.024). Six months after the intervention, participants who received FORNET reported significantly fewer depression symptoms (*t*(96) = 3.80, *p* < 0.001, Cohen's *d* = 0.77). Between‐group effect sizes were moderate. Only two clients in each group fulfilled the diagnostic criteria for Major Depression at baseline. While both participants in the FORNET group recovered, one additional participant fulfilled the criteria for Major Depression at the 6‐month follow‐up in TAU.

Regarding a reduction in the symptom severity of drug dependence, neither the interaction (time × treatment: *F*(1, 92) = 1.43, *p* = 0.235, $\varepsilon_{p}^{2}$ = 0.015, $\varepsilon_{G}^{2}$ = 0.010) nor the main effect (time: *F*(1, 91) = 3.07, *p* = 0.083, $\varepsilon_{p}^{2}$ = 0.032, $\varepsilon_{G}^{2}$ = 0.022) was significant. Accordingly, no significant between‐group difference was found 6 months after demobilization (*t*(96) = −0.16, *p* = 0.439). However, the remission rate of the DSM‐IV diagnosis Drug Dependence was substantially higher in the FORNET group; 24% of participants remitted in TAU versus 53% in FORNET.

Regarding the economic status, participants in the FORNET condition showed significantly better reintegration compared with those in TAU (*t*(96) = −2.77, *p* = 0.004, Cohen's *d* = 0.28). The mean differences for the connection with (para)military life were not significant (*t*(96) = −0.60, *p* = 0.276).

The covariate setting (DDR versus local reintegration camp) had no influence on primary (PTSD: *F*(1, 92) = 0.19, *p* = 0.666; appetitive aggression: *F*(1, 92) = 3.55, *p* = 0.063) or secondary (depression: *F*(1, 92) = 0.32, *p* = 0.576; drug dependence: *F*(1,92) = 1.13, *p* = 0.292) outcomes.

### Maintenance of Treatment Gains

As inferred in the flow of participants (Analysis II; Figure 1), we assessed the long‐term effects of FORNET with a subsample of ex‐combatants who demobilized in DS1 via DDR. Repeated‐measures ANCOVA, including 6‐month and 12‐month follow‐up assessments, revealed a significant interaction effect of time × treatment for PTSD (*F*(2, 20) = 7.18, *p* = 0.002, $\varepsilon_{p}^{2}$ = 0.935, $\varepsilon_{G}^{2}$ = 0.494). This effect was not significant for appetitive aggression (*F*(2, 20) = 1.49, *p* = 0.238, $\varepsilon_{p}^{2}$ = 0.748, $\varepsilon_{G}^{2}$ = 0.161). Pairwise comparisons 12 months after demobilization revealed significant mean differences between FORNET and TAU for PTSD symptom severity (Table 3), but not for appetitive aggression. Remission rates additionally confirmed the maintenance of treatment gains for PTSD: 100% were in remission in FORNET, whereas only one (20%) participant was in remission in TAU. Large effects were found for both secondary mental health outcome measures (depression: *F*(2, 20) = 7.88, *p* = 0.001, $\varepsilon_{p}^{2}$ = 0.940, $\varepsilon_{G}^{2}$ = 0.591; drug dependence: *F*(2, 20) = 2.92, *p* = 0.065, $\varepsilon_{p}^{2}$ = 0.854, $\varepsilon_{G}^{2}$ = 0.284). Symptoms of depression were lower in FORNET. Regarding drug dependence, mean differences were not significant at a 95% probability level but showed a trend towards significance (*p* < 0.10). Of those participants that fulfilled the diagnostic criteria of Drug Dependence and received FORNET, half were in remission at the 12‐month follow‐up, while in TAU, two additional participants fulfilled the diagnostic criteria. While in DS1 at 6 months after demobilization, no benefits of FORNET were found in between‐group comparison of the severity score of drug dependence, at 12 months, differences indicated decelerated benefits with *p* < 0.10.

### Comparison of Dissemination Stages 1 and 2

The 95% CIs of Cohen's *d* in DS2 compared with DS1 resulting from within‐group comparisons indicated comparable effects of FORNET. Regarding the primary outcome, the effect size for the reduction of PTSD symptom severity was large and equivalent in DS1 (*d* = 1.35) and in DS2 (*d* = 1.41); the upper and lower limits in DS2 (CI 0.81, 2.01) were located inside the 95% CI of Cohen's *d* in DS1 (CI 0.65, 2.04). The level of appetitive aggression was reduced moderately in DS2 (*d* = 0.70, CI 0.14, 1.25), but not in DS1 (*d* = 0.14, CI < 0, 0.67). For depression, the effect sizes were large and equivalent in the two dissemination stages (DS1: *d* = 1.14, CI 0.46, 1.81; DS2: *d* = 1.42, CI 0.82, 2.02). As there was still a major overlap of the CIs (86.2%), the reduction of drug dependence symptoms can also be considered equivalent in DS1 (*d* = 0.93, CI 0.26, 1.58) and in DS2 (*d* = 0.68, CI 0.13, 1.24). Similar results were found for between‐group comparisons, as shown in Table 3. Furthermore, the interaction terms for time × treatment × DS did not indicate different treatment effects for the first and second generation of counsellors (PTSD severity: *F*(1, 92) = 0.13, *p* = 0.724, $\varepsilon_{p}^{2}$ = 0.001, $\varepsilon_{G}^{2}$ 2 2 G < 0.001; appetitive aggression: *F*(1, 92) = 0.00, *p* = 0.955, $\varepsilon_{p}^{2}$ < 0.001, $\varepsilon_{G}^{2}$ < 0.001; depression severity: *F*(1, 92) = 0.00, *p* = 0.955, $\varepsilon_{p}^{2}$ < 0.001, $\varepsilon_{G}^{2}$ < 0.001; drug dependence: *F*(1, 92) = 0.55, *p* = 0.461, $\varepsilon_{p}^{2}$ < 0.006, $\varepsilon_{G}^{2}$ = 0.004). Finally, it is noteworthy that at the clinical level, the remission rates for the diagnosis of PTSD, Major Depression and Drug Dependence were in both dissemination steps higher compared with the control condition.

## Discussion

In a sample of adult ex‐combatants, we tested FORNET, a psychotherapeutic short intervention, which (a) addresses symptoms of pathological memory formations in the aftermath of both traumatic experiences and combat high and (b) was conducted by counsellors in a first and second dissemination generation. First, the results demonstrate that FORNET, compared with TAU, effectively reduced Posttraumatic stress. However, the level of appetitive aggression did not decline. Beneficial effects were found on secondary outcome measures, especially depression symptoms and Drug Dependence at the clinical level. Follow‐up assessments 1 year after the intervention suggested further treatment gains in the long term (assessed in DS1 only). Second, FORNET was successfully conducted by first‐generation versus second‐generation counsellors. Moreover, we figured that FORNET can effectively be implemented in the early demobilization process and in settings with ongoing conflict.

### Treatment Outcome

#### Posttraumatic Stress Disorder

Violent offenders with PTSD and trauma‐related disorders have an increased risk of not responding to NET when their offenses have not been explicitly addressed (Stenmark *et al*., 2014). Therefore, with FORNET, we have expanded evidence‐based NET to address the specific needs of offender populations, such as heightened levels of PTSD and aggression due to experience of traumatic stress and combat high (Elbert *et al*., 2012; Hermenau *et al*., 2013; Crombach and Elbert, 2014b).

With the present work, we respond to the lack of adequate field interventions in DDR programmes and (para)‐military samples in general. The results suggest that FORNET—implemented in the early demobilization process—effectively reduces trauma symptoms, even in a setting of ongoing severe conflict (Security Council Report S/2014/42). Importantly, the effect sizes were in accordance with other clinical trials that have assessed the effectiveness of more time‐consuming treatments conducted by clinical experts with (para)military samples (Steenkamp & Litz 2013; Zinzow, Britt, McFadden, Burnette, & Gillispie, 2012). For instance, Monson *et al*. (2006) applied an adapted version of CPT in a US veteran sample comprising 12 individual sessions that were delivered by doctoral‐level clinicians. Compared with a waiting list control group, they found a moderate effect on PTSD severity (Hedge's *g* = 0.67; intent‐to‐treat) and reported a remission rate of 30% 1 month after the treatment (no later follow‐up assessments were reported). In a sample of minor former child soldiers in DRC, McMullen *et al*. (2013) investigated the effectiveness of a 15‐session trauma‐focused Cognitive Behavioural Therapy (tf‐CBT) compared with a wait‐list control condition. They reported a moderate decline of PTSD symptoms ( $\varepsilon_{p}^{2}$ = 0.67). For a broader picture on the very heterogeneous studies with veterans in high‐income countries, see Steenkamp and Litz (2013) and Zinzow *et al*. (2012). More research is required.

#### Appetitive Aggression

Only minor effects were found for appetitive aggression without superiority of FORNET to TAU. In fact, this is in accordance with the two former trials (Hermenau *et al*., 2013; Crombach & Elbert, 2014b).

Notably, Crombach and Elbert (2014b) reported that former street children continued to rate violent acts as appealing, irrespective of their particular treatment condition—however—those who received FORNET did not act out violent acts as often as those in the control condition. It seems that memories of the ‘highs’ of combat keep ratings of violent acts elevated. However, this does not necessarily mean that former fighters—in the streets or in war—will behave more aggressively when returning to a civil setting, especially when the change in role is emphasized as in FORNET and defence‐driven violent reactions wane with the reduction of PTSD symptoms. The follow‐up in this investigation was not long enough to reliably measure the portion of the participants that rejoined an armed group. However, actual behavioural measures or indices of the number of crimes committed, as in the Crombach and Elbert (2014a) study, or biological measures may be needed to determine whether FORNET's module of role change together with the NET‐typical reprocessing of the memories of violent acts will actually change behavioural outcomes.

#### Trauma‐related Disorders

The effects on trauma‐related problems were promising: depression severity as well as the frequency of Major Depression was reduced significantly 6 and 12 months after demobilization. Moreover, the remission rates for Drug Dependence were higher in the FORNET groups of DS1 and DS2. The insignificant difference of the two groups regarding the symptom severity of drug dependence at the 6‐month follow‐up may suggest that FORNET, with its reduction of PTSD symptoms, primarily affects dependency in severe cases, where drugs may have been used compulsively to suppress trauma symptoms.

#### Reintegration

Reintegration outcomes seem to depend to a greater extent on political stability. In short, the conflict intensity in North Kivu increased in April 2012 with the invasion of M23 rebels into parts of North Kivu and peaked when the rebels entered North Kivu's capital, Goma, in November 2012 (3 months after the DS1 intervention). The conflict then declined with an increase of international support in May 2013, during the treatment phase of DS2. Accordingly, the connection with (para)military life within the FORNET group was higher in DS1 (when participants were released into an escalating conflict) compared with DS2 (when participants were released after the conflict's peak and after the implementation of additional international support). Moreover, the between‐group comparisons of economic reintegration were only significant in DS2 and at the 12‐month follow‐up for DS1. We consider this as evidence that the reduction of mental health problems will facilitate economic reintegration if the political situation leads to increasing stability. Given that the mean difference of the connection with (para)military life in DS2 was only significant at a 90% level and without correction for multiple testing, we consider this result as suggestive. Further investigation is required.

Ex‐combatants are trapped in a cycle of violence: intrusive memories and hypervigilance drive them towards outbursts of impulsive violence; simultaneously, many find violence as a drug‐like hedonic appeal and use it to control trauma‐related intrusive memories. In post‐conflict regions with less effective government authorities, this cycle has an especially powerful impact on the individual, as well as the family, the community and the stability of the country as a whole. It should be noted that the M23 rebel organization, at the time of its peak activity, was estimated to have a maximum of only 2500 members! It does not take many combatants to trigger extensive fighting in a failed state. Thus, addressing reactively driven violent outbursts and tendencies towards aggression in the course of the demobilization process is a worthwhile endeavor. With FORNET, our research group provides an effective psychotherapeutic intervention that can be implemented in stabilization programmes of various countries and can contribute to the large‐scale peace‐building mission. Its property of being easy to disseminate is an additional advantage.

### Dissemination of FORNET

Importantly, we also successfully passed down FORNET into a first and second generation of counsellors. A review of the global situation rapidly reveals the relevance of this finding: at present, there are 33 ongoing ‘new’ wars, i.e., wars that largely victimize the civilian population, including children. The majority of fatalities are civilians and at least three out of five soldiers have been recruited before the age of 18 years (own data) – summed up to an estimate of hundreds of thousands of children under arms (UNICEF). Many of these children have been abducted and threatened into submission, others find a way to escape poverty with joining an armed group, to defend their communities, or they join out of a feeling of revenge. With it, inevitably the starting point of a ceaseless cycle of violence is set for these children's lives. At a larger level, the probability of resolution for these wars is low, and even then, the risk of renewed outbreaks remains high (Glassmyer & Sambanis, 2008). One of the major sources of destabilization are former combatants, particularly those with poor reintegration (Banholzer 2014; Humphreys & Weinstein, 2004) and heightened levels of aggression (Hermenau *et al*., 2013). Further, the treatment gap is extremely high in LMIC, where the majority of civil wars are fought. The scarcity of adequately skilled personnel and easily disseminable, evidence‐based interventions are one of the main obstacles to treatment (Saxena *et al*., 2007; Tol *et al*., 2013). At this point, it is necessary to re‐design psychotherapeutic approaches that are effectively practiced in high‐income countries and to develop novel strategies to disseminate psychotherapeutic skills across generations of counsellors.

In two studies in Uganda where NET was utilized by local counsellors, the intervention successfully reduced PTSD and other trauma‐related symptoms (Ertl *et al*., 2011; Neuner *et al*., 2008). Later, Jacob *et al*. (2014) conducted a clinical trial assessing the dissemination of NET to a second generation of counsellors. In both dissemination stages, trauma symptoms of Rwandan genocide survivors were reduced significantly. Consistently, the results presented in this study demonstrate the potential for effective dissemination of FORNET conducted by the first and the second generation of counsellors as part of a demobilization programme.

The present findings demonstrate that FORNET effectively reduces PTSD and other trauma‐related symptoms in former members of (para)military groups. In addition, we demonstrated that FORNET can successfully be implemented in the demobilization process and that the intervention remains effective when disseminated across generations of counsellors without previous therapeutic experience.

## Limitations

The results are limited by the unnecessarily high number of participants that were lost to follow‐up, mainly in DS1, which was caused by the escalation of the conflict. This forced us to abandon matching dyads in the analysis to avoid further losses. Yet, compared with other studies in the eastern DRC (e.g., Bass *et al*., 2013) and considering the peak in conflict intensity, a high number of participants made the effort to travel back to the camp for follow‐up assessment, sometimes requiring a journey through unsafe territories. As the number of clients who were lost for the follow‐up assessment was similar in the treatment and control condition and the lost participants of the two conditions did neither differ in terms of sociodemographic variables nor the severity of their symptoms, we do not assume that the results are biased. With including participants that presented with only low symptom severities and excluding all participants that could not come for the follow‐up assessment (instead of using intent‐to‐treat for instance), we chose a more conservative approach that may rather have underestimated the treatment effects. Eventually, another trial would be required to answer the question; the probability of another insurgency in the follow‐up period is currently declining. Moreover, the assessment of the outcome variable aggressiveness has been suboptimal as we changed the instrument in course of the trial. Nevertheless, it is important having these results figured and taken into account for future operationalizations. In fact, the result mirrors the challenge to reliably assess aggression: an antisocial concept, in a highly social situation, namely, the diagnostic interview. Further research is required so that assessment methods can be refined to reflect behavioural changes more sensitively and to be less susceptible for subjective views of one's own aggression. Moreover, to investigate the clinical significance, future trials should include analysis like the Reliable Change Index, in addition to the reintegration indices that indicate the practical value of FORNET in the current trial. Finally, given the significant proportion of participants struggling with drug dependence symptoms, substance use disorders deserve more consideration in future clinical trials.
